# Role of Polymorphisms of NKG2D Receptor and Its Ligands in Acute Myeloid Leukemia and Human Stem Cell Transplantation

**DOI:** 10.3389/fimmu.2021.651751

**Published:** 2021-03-30

**Authors:** Alena Machuldova, Monika Holubova, Valentina S. Caputo, Miroslava Cedikova, Pavel Jindra, Lucie Houdova, Pavel Pitule

**Affiliations:** ^1^ Laboratory of Tumor Biology and Immunotherapy, Biomedical Center, Faculty of Medicine in Pilsen, Charles University, Pilsen, Czechia; ^2^ Department of Haematology and Oncology, University Hospital Pilsen, Pilsen, Czechia; ^3^ Hugh & Josseline Langmuir Center for Myeloma Research, Center for Hematology, Department of Immunology and Inflammation, Imperial College London, London, United Kingdom; ^4^ Cancer Biology and Therapy Laboratory, School of Applied Sciences, London South Bank University, London, United Kingdom; ^5^ NTIS, Faculty of Applied Sciences, University of West Bohemia, Pilsen, Czechia; ^6^ Department of Histology and Embryology, Faculty of Medicine in Pilsen, Charles University, Pilsen, Czechia

**Keywords:** natural killer group 2 member D, MICA, MICB, ULBP, hematopoietic stem cell transplant, acute myeloid leukemia, polymorphism

## Abstract

Natural killer cells possess key regulatory function in various malignant diseases, including acute myeloid leukemia. NK cell activity is driven by signals received through ligands binding activating or inhibitory receptors. Their activity towards elimination of transformed or virally infected cells can be mediated through MICA, MICB and ULBP ligands binding the activating receptor NKG2D. Given the efficiency of NK cells, potential target cells developed multiple protecting mechanisms to overcome NK cells killing on various levels of biogenesis of NKG2D ligands. Targeted cells can degrade ligand transcripts *via* microRNAs or modify them at protein level to prevent their presence at cell surface *via* shedding, with added benefit of shed ligands to desensitize NKG2D receptor and avert the threat of destruction *via* NK cells. NK cells and their activity are also indispensable during hematopoietic stem cell transplantation, crucial treatment option for patients with malignant disease, including acute myeloid leukemia. Function of both NKG2D and its ligands is strongly affected by polymorphisms and particular allelic variants, as different alleles can play variable roles in ligand-receptor interaction, influencing NK cell function and HSCT outcome differently. For example, role of amino acid exchange at position 129 in *MICA* or at position 98 in *MICB*, as well as the role of other polymorphisms leading to different shedding of ligands, was described. Finally, match or mismatch between patient and donor in NKG2D ligands affect HSCT outcome. Having the information beyond standard HLA typing prior HSCT could be instrumental to find the best donor for the patient and to optimize effects of treatment by more precise patient-donor match. Here, we review recent research on the NKG2D/NKG2D ligand biology, their regulation, description of their polymorphisms across the populations of patients with AML and the influence of particular polymorphisms on HSCT outcome.

## Introduction

Acute myeloid leukemia (AML) is an aggressive malignancy originated from a myeloid lineage of bone marrow cells with median overall survival of 8.5 months and 24% 5-year overall survival according to National Cancer Institute in the USA (1, 2). Most patients with AML achieve complete remission after chemotherapy treatment, but relapse is almost inevitable and often indicates the appearance of treatment-resistant clones (3). Carrying specific gene mutations enable drug-resistance, survival, and uncontrolled proliferation. However, for the disease progression, AML cells also need to escape immune system control. Healthy immunosurveillance should eliminate AML cells by using two main effector cells types that play pivotal and complementary role in this mechanism - T lymphocytes and natural killer (NK) cells. Unlike T cells, whose function depends on the recognition of “non-self” peptides presented on HLA molecules, NK cells recognize cells with or without the altered level of HLA molecules and thus can recognize transformed cells that hide these molecules as a mechanism of escape T-cells (4). Besides direct attack targeted to transformed cells, NK cells also compete with myeloid leukemic blasts to colonize the bone marrow niche and to adhere to bone marrow fibroblasts, preventing myeloid blasts from proliferation (5).

Interaction between AML and NK cells exists in both directions, as AML cells use multiple mechanisms to protect themselves, including modification of NK cells in patients. Individuals with AML have low levels of cytotoxic NK cells, usually combined with decreased expression levels of activating receptors, for example, NKp46 (6), NKp30 (7), NKG2D (6) or DNAM-1 (8, 9). In contrast, inhibitory receptors tend to be increased, for example a decreased DNAM-1 is being associated with increased expression of inhibitory receptors TIGIT and/or TACTILE (9). Another inhibitory receptors implicated in NK cells’ tolerance to the AML blasts are inhibitory KIR, NKG2A, CD158b, and LIR-1 (6, 10). In addition, AML cells express a high amount of soluble activating receptors’ ligands responsible for NK cells silencing through downregulation of their receptors by chronic exposure causing exhaustion of NK cells (11).

To overcome immunosurveillance impairment caused by malignant cells, hematopoietic stem cells transplantation (HSCT) is an essential treatment option for patients with relapsed AML. NK cells have a crucial role in the success of HSCT by affecting host, graft, and residual leukemic cells. The graft-versus-leukemia (GvL) effect of NK cells was already described in 1986 (12). In addition, NK cells decrease the incidence of graft-versus-host disease (GvHD) by reduction of antigen-presenting cells, and elimination of host T cells, preventing graft rejection (13).

To take full advantage of their potential, it is crucial to understand the mechanisms of ligand-receptor functions and to know better the parameters, which can influence the outcome of HSCT. Balance of NK cells reactivity depends on the combination of signals transduced from inhibitory and activating receptors and their ligands present on “examined” cells. Main and broadly investigated inhibitory and activating receptors of NK cells are KIR receptors which recognize HLA class I-bearing targets, while activating natural cytotoxic receptors (NCR), namely NKp30, NKp44 and NKp46, DNAM-1 and NKG2D recognize non-HLA molecules and ligands expressed *de novo* on “stressed” cells (14–17).

This review focuses on activating NKG2D receptor, its ligands, their regulation, and on the role of their polymorphism in AML patients and dependency of the polymorphism of NKG2D and its ligands on HSCT outcome.

## NKG2D Receptor

NKG2D (encoded by the *KLRK1* gene – killer cell lectin-like receptor subfamily K, member 1) is a C-type lectin receptor present on the surface of natural killer (NK) cells, γδ T cells, CD8+ T cells and CD4+ T cells (18–20).

*KLRK1* is composed of 10 exons (exons 1A, 1B, and 2–9) and 9 introns, with exons 2–4 encoding intracellular/transmembrane domain and exons 5–9 encoding the ligand-binding outer domain, which is exposed into the extracellular space (21).

NKG2D receptor recognizes and binds multiple ligand families, and upon ligand’s engagement it interacts with adapter dimer DAP10, which triggers activation signal leading to cell-mediated cytotoxicity (degranulation), co-stimulation of cytokine production, playing an important role in the tumorous and infected cells elimination (18, 22, 23).

Although *NKG2D* shows strong evolutionary conservation, two different haplotype blocks have been described. Haplotype block Hb-1 contains two alleles called *LNK1* (low-activity related) and *HNK1* (high-activity related), in three haplotype combinations – *LNK1/LNK1*, *HNK1/HNK1*, and *LNK1/HNK1*. For haplotype block Hb-2, the situation is similar with haplotypes containing *LNK2* and *HNK2* alleles (24).

From a clinical perspective, *HNK1/HNK1* and *HNK2/HNK2* haplotypes seem to be associated with lower cancer risk than *LNK1/LNK1* and *LNK2/LNK2*, respectively (24). NKG2D receptor haplotypes also show a significant impact on transplantation outcomes. Patients with standard-risk hematology malignancy (AML and acute lymphoblastic leukemia, ALL in first complete remission, malignant lymphoma in complete remission, chronic myeloid leukemia, CLL in chronic phase and any status of a myelodysplastic syndrome) undergoing HSCT with HNK1 haplotype donor have lower transplantation-related mortality and better overall survival (difference in 5-year overall survival of 73% vs. 49%, p=0.01) (18). NKG2D haplotype also represents a candidate biomarker for the prediction of treatment-free remission, described currently in patients with CML treated by dasatinib, where patients with *HNK1/HNK1* haplotype achieved molecular response faster than patients with other haplotypes (25).

As mentioned in the *Introduction*, AML cells can modify the expression of activating receptor of NK cells, including the NKG2D receptor, as described by Hilpert et al. in 2012 (26). Khaznadar and colleagues investigated AML patients, dividing them into two groups according to clinical outcome. The group with deficient NK cell profile (NK-DP), reduced expression of NKG2D, DNAX accessory molecule-1, NKp46 and IFN-γ had a higher risk of relapse, while the group with NK cell-high profile (NK-HP) had a significantly lower risk of relapse and better median overall survival (HR 0.66, 95% CI 0.44-0.99) (27).

## NKG2D Ligand

NKG2D ligands in human can be divided into two families - MIC (MHC class I-related chain) family and ULBP/RAET (HCMV Unique Long 16-binding protein/Retinoic acid early transcript) family. Both are distant HLA class I homologues but do not associate with β-2 microglobulin and have no known role in antigen presentation (26).

NKG2D ligands are called stress-ligands as their presence is stimulated predominantly in damaged, virally infected, or tumorous cells (27). Besides of these stimuli, these ligands can be upregulated by standard cell-stress conditions including heat shock (28), oxidative stress (29), and ionizing radiation (28, 30).

NKG2D ligands are also expressed on healthy conditions, particularly on proliferative cells like embryonic cells (31), myeloid progenitors (32), normal intestinal epithelial cells (33), or cells of repairing tissue (34). The mechanisms protecting these cells against NK cell attack are not fully known. It seems that on healthy cells, NKG2D ligands alone is not sufficient signal to trigger NK cell activation (35). The intracellular localization of NKG2D ligand is an additional observed protecting mechanism causing ligand’s inaccessibility to the receptor (33, 35). On the other hand, the presence of NKG2D ligands on immune cells also plays an important role in regulating immune responses (36).

The polymorphism of NKG2D ligands genes affects susceptibility to different diseases (37), disease severity (38), transplantation outcome (organ or HSCT), and serves as a risk factor (39) and/or as protective factor for cancer (40).

### MIC Family

MIC family comprises 7 genes, from which only two are expressed (*MICA* and *MICB*) and an additional five being considered as pseudogenes (*MICC, MICD, MICE, MICF*, and *MICG*) (41).

**Figure 1:**
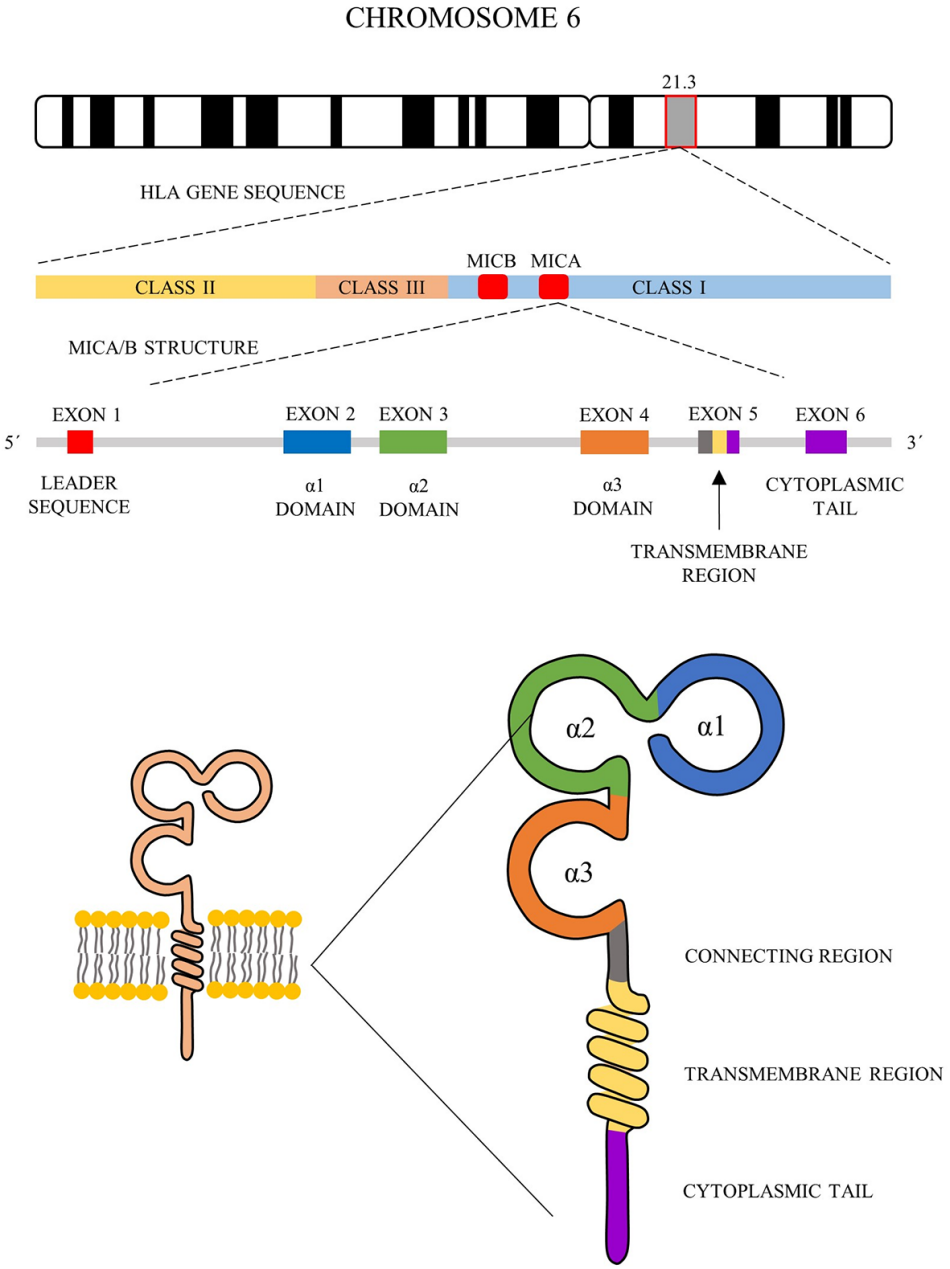
Schematic representation of MICA and MICB structure. *MICA* and *MICB* comprise 6 exons and 5 introns (middle) - exon 1 encodes leader sequence, exons 2 - 4 encode three extracellular domains α1, α2 and α3, exon 5 encodes the transmembrane region and exon 6 encodes cytoplasmic tail (bottom).

Both *MICA* and *MICB* contain 6 exons and 5 introns - exon 1 encodes leader sequence, exons 2 - 4 encode three extracellular domains α1, α2, and α3, exon 5 encodes the transmembrane region and exon 6 encodes the cytoplasmic tail. The majority of polymorphisms of *MICA* and *MICB* alleles are concentrated on exons 2, 3, 4, and 5, predominantly within α2 and α3 domains (**Figure 1**) (41, 42).

**Figure 2:**
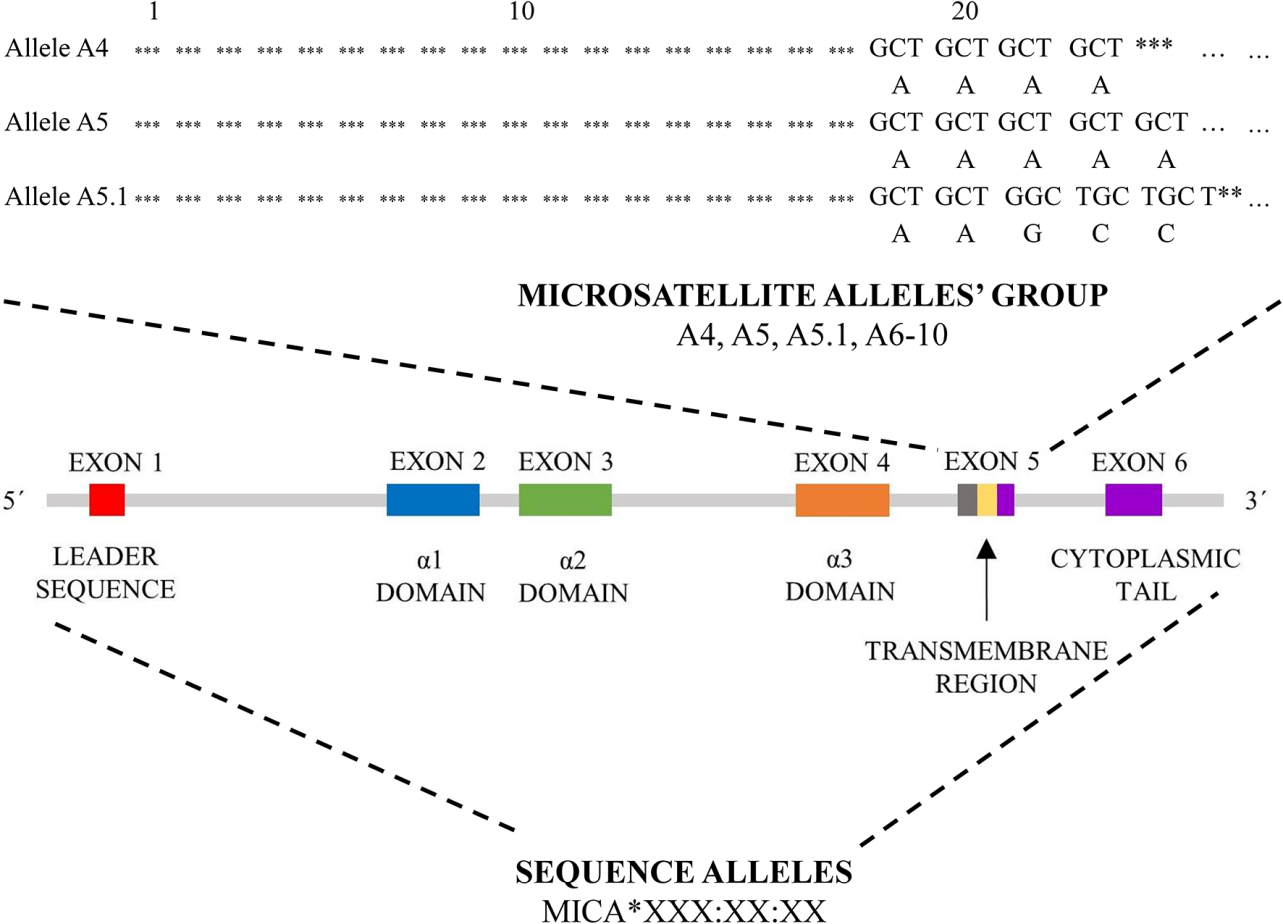
MICA nomenclature. Two types of MICA nomenclature are shown. Top panel, based on microsatellite repeat (GCT_n_) within exon 5, which encodes a different number of alanines (A4-10). Bottom pannel, based on sequence of exons 2, 3, 4 and 5 and its nomenclature correspond to HLA nomenclature (MICA*XXX : XX:XX).

In the current literature, there are two different nomenclature approaches for MICA allele description (summarized in **Figure 2**).

The first approach is based on a sequence of exon 5, called exon 5 (EX5) microsatellite alleles’ group, and corresponds to a repeated sequence of 4 to 10 Ala (GCT) codons within this exon, with alleles named *A4, A5, A5.1, A6-10. A5.1 MICA* alleles are specific by having additional guanine nucleotide insertion between codons 20 and 21, resulting in a premature stop codon at codon 41 (43). This truncated protein lacks the cytoplasmic tail encoded by exon 6 (41, 44). This difference is translated into the form of anchorage of the final protein to the cell membrane. Most of *MICA* alleles are transmembrane-anchored glycoprotein with a cytoplasmic tail; A5.1 alleles create glycophosphatidylinositol (GPI)-anchored glycoproteins (45). To date, it is not clear if other microsatellite alleles (*A4, A6-A10*) could encode similar truncated proteins too. This feature seems to arise as a consequence to cytomegalovirus’ (CMV) immunoevasin UL142 (46). This immunoevasin enables the immune escape of the infected cells by retaining protein product of *MICA* alleles in the Golgi apparatus while not affecting truncated proteins encoded by *MICA A5.1* alleles (46, 47).

The second nomenclature approach is based on the gene sequence, but not always all exons have been sequenced. The sequence alleles nomenclature (for example MICA*008:01:09) is similar to the HLA World Health Organization nomenclature, comprising gene name (*MICA* or *MICB*), separator (*), allele group (for example *008), field separator (): specific MICA protein (two-digit format, for example, 01) and next closer specification as described at hla.alleles.org/nomenclature/naming.html (48). This nomenclature approach is used not only for *MICA* but also for *MICB* alleles (48). To date 223 *MICA* sequence alleles encoding 104 proteins and 138 *MICB* alleles encoding 37 proteins have been described (release June 2020) (48).

According to the large study of 1.2 million donors with German descent, the most common MICA allele in Caucasian (German) population is MICA*008 (*A5.1*) with a frequency of 42.3% followed by MICA*002 (*A9* microsatellite allele) with 11.7%, and MICA*009 (*A6* microsatellite allele) with 8.8%. The most common *MICB* allele in the same population is MICB*005 with a frequency of 43.9% followed by MICB*004 with 21.7% and MICB*002 with 18.9% (49). Taking another populations into account, MICA*008 is the most frequent MICA allele worldwide with MICA*002, MICA*009, MICA*010 and MICA*004 taking next places varying according to inter-ethnic variability (49–51). Also, *MICB* results across the population correspond to the German study, i.e., most frequent is MICB*005 and followed by MICB*002, MICB*004, MICB*014, and MICB*003 (52).

### ULBP Family

All 10 ULBP family members are officially named *RAET1* genes and are orthologs of the mouse Raet1 genes (53). Six genes of them are expressed (*ULBP1 - 6*), while 4 are pseudogenes (53).

*ULBP1 (RAET1I), ULBP2 (RAET1H), ULBP3 (RAET1N), ULBP4 (RAET1E)* and *ULBP6 (RAET1L)* have 4 exons while *ULBP5 (RAET1G)* comprises 5 exons. Exon 1 encodes leader sequence, exons 2 and 3 define the α1 and α2 domains, and exon 4 encodes a hydrophobic sequence, both for GPI anchor region and transmembrane region with the cytoplasmic tail (53–56). ULBP2 and ULBP5 can form both transmembrane-anchored and GPI-anchored form, ULBP1, ULBP3 and ULBP6 are only GPI-anchored and ULBP4 forms just transmembrane-anchored protein (**Table 1**) (57, 58). Exon 5 seems to encode for the extended cytoplasmic domain (ULBP5) or is non-coding (other ULBPs) (54–56). Compared with MIC, ULBP proteins lack α3 domain (53), which seems to have no effect on NKG2D binding (53), but it probably precludes CD8 (T cells co-receptor) binding (59).

**Table 1:** Human NKG2D ligands.

Ligand name	Alternative name	Number of exons	Anchorage type
MICA	PERB11.1	6	TM/GPI
MICB	PERB11.2	6	TM
ULBP1	RAET1I	4	GPI
ULBP2	RAET1H	4	TM/GPI
ULBP3	RAET1N	4	GPI
ULBP4	RAET1E	4	TM
ULBP5	RAET1G	5	TM/GPI
ULBP6	RAET1L	4	GPI

TM, transmembrane type; GPI, glycosylphosphatidylinositol-linked type.

*ULBP/RAET1* genes seem to be less polymorphic than MIC genes, although this could be caused by a lower number of sequenced samples (52). Four out of the six *ULBP* genes have been found to be polymorphic in exons 2 and 3, coding extracellular part of the protein. So far no polymorphism has been described in *ULBP1* and *ULBP3* within exons 2 and 3 (52). The most polymorphic gene is *ULBP4* with 11 known alleles (52), followed by *ULBP6* with 7 known alleles, *ULBP2* with 6 alleles and *ULBP5* with3 described alleles (52, 60). Currently, *ULBP* polymorphism has been studied in 223 Euro-Caucasoid, 60 Afro-Caribbean, 52 Indo-Asian individuals (61), Kolla South American Indians (60) and Thais (62), a broader sampling is required to achieve a true picture of *ULBP* polymorphism. For the Caucasian population, the most common *ULBP4* allele is *002, while for Thais it is *001, and for Kolla Indians it is *003 (60). Similarly, *ULBP6* alleles frequency also differs between populations, Caucasians having *003 most often, Thais *001 and Kolla Indians *002 (60). The variability in the frequency of ULBP alleles among individual populations seems to be associated with differences in life conditions and contact with unique pathogens (60).

## Regulation of NKG2D Ligands

**Figure 3:**
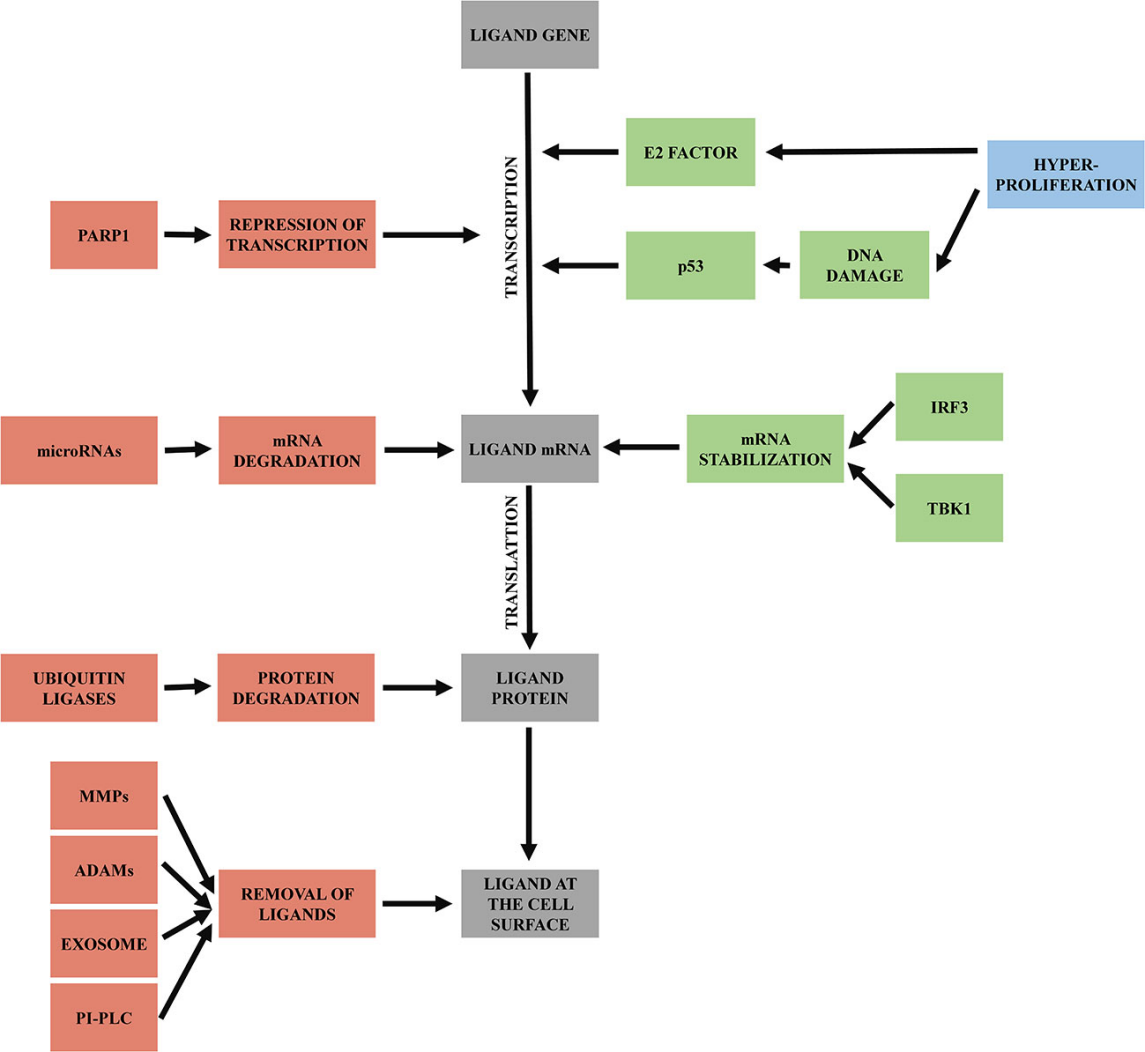
Regulation of NKG2D ligands. Mechanisms triggering transcription of ligand genes and mRNA stabilization are labeled as green. Processes leading to mRNA or protein degradation or removal of ligands from cell surface are labeled as red.

As mentioned above, NKG2D ligands are stress-associated molecules upregulated on damaged or transformed cells to attract immune cells, especially NK cells. Their expression is regulated by numerous pathways and signals on multiple levels of biogenesis - on transcriptional, translational, and post-translational level (**Figure 3**).

In malignant cells, upregulation of NKG2D ligands expression is induced initially by hyperproliferative state occurring during early tumorigenesis. Hyperproliferation activates E2 transcription factor, which induces NKG2D ligands’ transcription (34). Hyperproliferative state could also trigger DNA damage response (63), and so activate p53 (64). Activated p53 then enhances transcription of NKG2D ligands (65).

NKG2D ligands’ mRNA usually has a very short half-life; it is rapidly degraded and needs to be stabilized for translation. Besides the increased transcription, DNA damage response also plays a role in the mRNA stabilization *via* signaling protein TBK1 and transcription factor IRF3, which induce conditions that help to increase the half-life of NKG2D ligands transcripts (66).

In contrast, faster degradation of NKG2D ligand transcripts can be induced by specific microRNAs that bind to their 3’ untranslated region and repress their translation (67). MicroRNA regulates stress ligands overexpression, as only when *MICA* and *MICB* mRNA exceeds the microRNA repression, the NKG2D ligands are expressed on the cell surface. Overexpression of microRNAs can help tumors to downregulate NKG2D ligands expression and then escape the immune system (68).

Another mechanism leading to the reduced levels of surface NKG2D ligands is *via* poly-ADP-ribose polymerase 1 (PARP1) mediated repression of transcription. This mechanism seems to be specific for leukemia stem cells and enables them to escape immune surveillance and later to cause relapse of disease (69). This opens the possibility to use PARP inhibitors for AML patient (70), an approach that the first clinical trials are currently assessing (71–73).

At the protein level, NKG2D ligands are regulated by various post-translational modifications. First, described in MULT1 ligand (mouse homolog of human ULBP1), is ubiquitin-dependent degradation (74). Ubiquitin modifications occur primarily on lysine residues of target proteins (75). The presence of multiple lysins in the cytoplasmic tail of MICA, MICB and ULBP5 (RAET1G) suggests that this regulation could also exist for these human ligands (76). It has been shown, for example, that KSHV (Kaposi’s sarcoma-associated herpesvirus) uses E3 ubiquitin ligase K5 to downregulate cell surface expression of MICA and MICB (77).

A crucial system of NKG2D ligands regulation is the production of soluble variants of the NKG2D ligands by various mechanisms - co-transcriptional alternative splicing, post-translational ligand shedding, exosome ligand excretion and phospholipase C (PI-PLC) cleavage for ligands anchored by GPI. Shed ligands passively block NKG2D receptor on the NK cell, which is then internalized into the cell and degraded, leading to impairment of its function (78). This mechanism helps cancer cells to escape immune surveillance by blocking NKG2D and consequently NK cell activity. Another benefit of released ligands for cancer cell is that this cell became less visible for NK cells due to the lower NKG2D ligands’ concentration on its surface (79).

Co-transcriptional regulation by alternative splicing can also lead to soluble forms of NKG2D ligands, and has been described for ULBP4 (55) and ULBP5 (54). In RAET1G (ULBP5) it creates a product RAET1G2, where alternative splicing in exon 4 caused a frameshift and premature termination of the protein sequence and thus led to the soluble form of RAET1G (ULBP5) (54). The next example was described in ULBP4 (RAET1E) with a product of spliced variant called RAET1E2 where a stop codon was placed within intron between exons 3 and 4, producing a shortened form of ULBP4 (RAET1E) (80).

First, ligand shedding is mediated by two proteases families, “a disintegrin and metalloproteinase” (ADAMs) family (81) and matrix metalloprotease family (MMPs) (82, 83). From the ADAM proteases family, ADAM9, ADAM10 and ADAM17 have been shown to be active in shedding of MICA, MICB and ULBP2 ligands (84). Matrix metalloproteases are able to shed ULBP2 (83) or MICA (82, 85). Polymorphism of some NKG2D ligands can bypass the shedding process; for example, MICA*008 is bound to the membrane through GPI anchor, makes this allele resistant to protease-mediated cleavage (45).

The second way for cancer cells to remove NKG2D ligands at the protein level is the use of constitutive production and release of endosome-derived vesicles called exosomes, which eliminate active proteins or microRNAs from cells. The NKG2D ligands are expressed and carried on the surface of tumor exosomes and released into the environment (86). The NKG2D ligands released *via* exosomes have a proven effect on the downregulation of NKG2D and desensitization of cytotoxic cells (87, 88).

The last mechanism which cells can use to cut off their ligands is using cleavage *via* phospholipase release (PI-PLC), described in GPI anchored ULBP1 (RAET1I), ULBP2 (RAET1H), ULBP3 (RAET1N) and ULBP6 (RAET1L) (89). This mechanism is described on gastric tumor cells (89) and needs to be confirmed for other types of cancer.

The way of ligands shedding, either by protease cleavage or by exosomes, is most likely dependent on ligands attachment to the cell membrane. GPI-anchored *MICA* alleles vs. other transmembrane attached MICA alleles could serve as a model – protein product of MICA*008, GPI-anchored NKG2D ligand, is not usually cleaved by proteases but is released by exosomes (90). On the other hand, other transmembrane-attached MICA, MICB, or ULBP are primarily released by cleavage (81, 83, 91). Some ligands, for example ULBP2, can be released by both ways (88).

Solubilization of NKG2D ligands thus can have severe consequences for cancer patients, as lower cell-surface NKG2D ligands concentration (83) and presence of soluble NKG2D ligands in sera, very often, correlate with higher tumor stage and poor prognosis (84). For example, metalloproteinase ADAM10 is highly expressed in malignant pleural mesothelioma (92), prostate cancer (93) or in oral squamous cell carcinoma (94), which makes all of those tumor types able to escape immune surveillance by NKG2D ligands shedding.

In general, solubilization of MICA is a widespread mechanism of NK cell escape in malignant diseases. Holdenrieder et al. described an increased level of soluble MICA in lung cancer, colorectal and other gastrointestinal cancers, breast cancer, ovarian cancer, other gynecologic cancers, renal cancer, and prostate cancer as mentioned above (79). Paschen et al. described increased levels of sMICA also in melanoma (95), while in hematological malignancies, a higher level of sMICA and sMICB was described in multiple myeloma (96) and various leukemias (97, 98).

## NKG2D Ligands and Acute Myeloid Leukemia

As described above, multiple mechanisms enable tumors to escape immune surveillance with ligand shedding being one of the most important. In relation to AML, in 2003, a seminal work of Salih et al. investigated AML blasts and found various NKG2D ligands expressed on these cells, but they also demonstrated that patients with AML had significantly elevated levels of soluble MICA and MICB in comparison with healthy donors (99). This observation was confirmed nine years later by Hilpert et al., who discovered that 70% of AML blasts were positive for at least one NKG2D ligand (with 15% of patients having leukemic cells expressing even four or five different NKG2D ligands), and in addition, 100% of patients with AML in this study had detectable serum levels of NKG2D ligands with MICA being the one most often detected in the sera (97). In AML patients, also complete absence of surface expression of NKG2D ligands was described, and it is believed to be a consequence of malignant cell development (32).

In addition to shedding and no presence of NKG2D ligands on the cell surface, polymorphisms of NKG2D ligands may play a role in AML development, although data are still too sparse to make clear conclusions. Currently, there are only two studies addressing the relationship between *MICA* polymorphisms and leukemia, including acute myeloid leukemia, work of Luo et al. focused on people of Han nationality of Southern China (100) and work of Baek et al. on Korean patients (101). There is a need to extend this type of studies to a wider population to allow for significant conclusions.

Luo et al. described differences between patients with leukemia and healthy ethnically matched controls. According to their observation, homozygotes with microsatellite alleles *A5* and allele MICA*010 have increased risk for developing leukemia (specifically for AML, the ratio of frequency was 35.9% in patients with AML vs. 17.6% in control samples). This makes *MICA A5* a risk factor for AML. Ji et al. in their meta-analysis focused on multiple types of cancer in multiple populations, described alleles *A5* as protective factor for its carrier (40), which seems contradictory with AML results of Luo et al. It is necessary to further study the correlation between alleles *A5* as a positive or negative risk factors across multiple populations and specific cancers. Alleles *MICA A5.1* (including the most frequent allele MICA*008) frequency was decreased in heterozygous leukemic patients, described only on lymphocytic leukemia, with data for myeloid leukemia, unfortunately, missing (100).

The other study with data from Korean patients included 324 patients with AML, ALL, and MDS, with 172 AML samples. In AML patients, the authors observed a higher frequency of *MICA A9* alleles than in the control group (34.9% vs. 22.0%). Although alleles *A9* can be found on a transcript level more frequently in AML patients, presence of protein product is often reduced on the cell surface by release or shedding of the ligand from AML cells and rendering tumor cells less detectable by NK and T cells (101). Similarly to Han population described above, *MICA A5.1* alleles were found with lower frequency in AML patients than in controls (25.0% vs 38.0%) (101).

Only a little is known about the relationship between of AML and *MICB*. Baek et al. observed a difference between patients and controls only within one *MICB* allele, MICB*005:03, with lower frequency in AML patients (2.9% vs. 10.5%) (101).

In the case of *ULBP*, only one study analyzed *ULBP* polymorphisms and their distribution among patients with hematological malignancies and among their donors with no difference in alleles frequency observed. Therefore, *ULBP* cannot currently be used as genetic determinants for the risk of developing a hematological malignancy (61, 102). On the other hand, although Mastaglio et al. were not assessing polymorphisms, they described *ULBP1* expression on AML blasts correlating with improved 2-year overall survival, relapse-free survival, and reduced relapse (103).

## NKG2D Ligands and Transplantation Of Hematopoietic Stem Cells

Transplantation of hematopoietic stem cells is a standard treatment of multiple hematology malignancies, as well as non-malignant diseases (104). The aim of transplantation of HSCT lies in the graft’s ability to react against residual cancer cells present in the patient, the effect known as graft-versus-leukemia (GvL) response (105, 106). Unfortunately, HSCT has several side effects and most patients experience some serious complications – acute-graft-versus-host disease (aGvHD), chronic GvHD (cGvHD) and/or relapse (107, 108). These complications also negatively impact patients’ quality of life and increase mortality.

GvHD can be described as an exaggerated manifestation of inflammation based on the interaction between donor lymphocytes and foreign (i.e., patient’s) antigens (109). The incidence of GvHD (both types) ranges between 40 to 60%, with mortality around 15% (109). Chronic GvHD is a major cause of mortality in long-term survivors of HSCT (110), and acute GvHD is the second leading cause of death after HSCT (111).

From a pathophysiology perspective, acute GvHD is caused by a conditioning regimen that damages target tissue (112). This damage leads to a cytokine storm which activates antigen-presenting cells (APC) from the patient (and later also donor’s APC), that are recognized by donor’s T cells (113). During the second phase, donor’s T cells are activated by interaction with APC, and they proliferate and differentiate into helper T cells Th-1, cytotoxic T cells and Th-17/Tc-17 (114). These activated T cells then produce additional cytokines, such as IL-2, which promotes further activation of T cells and also triggers NK cell responses (115). The last phase is based on escalation of inflammation resulting in end-organ damage (109). The mortality risk depends on the stage and grade of aGvHD (111). Among the most affected organs are the upper and lower gastrointestinal tracts, liver, and skin. Grading depends on the combination of damage of these organs (111). A stricter definition can be found in NIH consensus criteria from 2014 (116).

Chronic GvHD also consists of three phases, and its trigger is tissue damage caused by aGvHD, another cytotoxic injury or infection (117). This damage activates innate immune cells but also non-hematopoietic cells. The second phase is based on the overreaction of the adaptive immune system going hand in hand with the reduction of immune cell regulators, such as regulatory T-cells, causing upregulation of helper T cells Th-1, Th-17 and in contrast to aGvHD, also Th-2 (117). Phase three consists in abnormal tissue repair (117). Clinical manifestations can be similar to autoimmune disorders, it manifests in oral mucosa, for example, lichen or planus, it attacks eyes, causing, for example, keratoconjunctivitis secca or uveitis, and it targets skin, soft tissues, or inner organs (liver, lung disease, gastrointestinal tract or CNS) (110).

Older definition (currently seldom used) required onset of symptoms within 100 days for aGvHD and later onset for cGvHD. This is currently replaced by NIH definition, which is based on clinical manifestations rather than on the time of the onset alone (116).

As both GvHD types are potentially dangerous and life-threatening, all efforts are directed to avoid GvHD completely or to suppress GvHD manifestation (106). This can be achieved by a complete match of patient’s and donor’s HLA molecules (10 out of 10) (118). However, the full match can lead to a lower GvL reaction. The key clinical issue is then minimization of GvHD and maximization of GvL (119). Some mismatches between donor and patient in HLA, such as some HLA-DPB1 or HLA-Cw, can decrease the risk of relapse, which is caused by GvL reaction of T cells while not increasing the risk of severe GvHD (119). But leukemic cells can also hide HLA molecules and then escape T cells surveillance. In this situation, NK cells can be fundamental to eliminate tumorous cells because under standard circumstances, NK cells are inhibited by HLA molecules. When HLA molecules are missing, NK cells do not receive an inhibition signal and wait to be activated. Activating signal can come from NK cells-related activating KIR ligand-receptor in case of a match between patient and donor (120), or it can come from activating NKG2D receptor, where ligands match, mismatch or even their polymorphisms can play a crucial role (121). But there are still many unanswered questions, like which specific effect individual polymorphisms play? And do we know some particular polymorphisms which could influence HSCT outcome, or if match or mismatch in NKG2D ligands between donor and recipient influence HSCT?

### MICA and HSCT Outcome

Theoretically, the mismatch between donor and patient in *MICA* should lead to impaired NK cell, and T cell activation as the ligand is not recognized by the receptor. The clinical effect in patient with mismatched donor then should show a lower GvL effect worsening overall survival, and on the other hand, a lower risk of GvHD could be expected.

Indeed, Fuerst et al. described a higher risk of relapse (lower GvL effect) in the donor-recipient mismatch of *MICA*. His study was focused on match/mismatch in one amino acid – methionine or valine – at position 129 of *MICA*, called *MICA-129 Met* or *Val* (122). Based on his data, *MICA-129* mismatch can also lead to higher non-relapse mortality overall, meaning mostly aGvHD (122). According to Parmar and coworkers, aGvHD in patients with mismatched MICA is triggered by αβ T cells. They speculate that these cells’ response to MICA allo-antigens is similar to mismatched HLA antigens present on APC (123). It is worth mentioning that overall survival (OS) rates were lower in the case of *MICA-129* mismatch, but OS rates were similar for matched and mismatched *MICA* pairs at allele level (122). We can then speculate that it is amino acid at position 129, which plays the most important role in HSCT outcome and *MICA* match/mismatch. Focusing on *MICA* alleles generally, it has been described multiple times that donor-recipient match leads to a lower risk of aGvHD (122–124). We can use similar logic also to cGvHD, whose incidence is increased in transplant pairs mismatched for *MICA* (124). Compared to GvHD occurrence, data regarding the disease relapse is not consistent across the literature. Unlike Fuerst, Carapito et al. described lower risk of relapse in transplant pairs mismatched for MICA (124). A similar result regarding the relapse and mismatch in *MICA* between patient and donor was described by Parmar et al. (123). In this study focused on myeloid leukemias, 3-year cumulative incidence of relapse in patients with *MICA* mismatched vs *MICA* matched graft was higher for patients with a matched graft (20% for mismatched versus 35% for matched graft) (123). Here authors correlated stronger GvHD with a lower risk of relapse and thus higher GvL effect, also corroborated by other studies (125, 126).

The impact of the type of amino acid at position 129 (*MICA-129Met* or *MICA-129Val*) was described to play a role in HSCT not only from match/mismatch point of view. *MICA-129Met* isoform binds NKG2D receptor with higher affinity than *MICA-129Val* isoform, which leads to stronger activation of NK cells – it stimulates NK-cells mediated cytotoxicity more effectively, it triggers stronger IFNγ release and a faster activation of CD8+ T cells (121). On the other hand, this stronger and faster reaction leads to quicker NKG2D downregulation and reduction of effectivity of NK cells followed by lower activation of CD8+ T cells, a phenomenon called exhaustion of NK cell activity (121). On the other hand, *MICA-129Val* isoform induces weaker but longer-lasting NK cell reactivity (121). Consistent with this knowledge is the fact that homozygous carriers of *MICA-129Met* alleles have an increased risk to experience acute GvHD (121), and homozygous carriers of *MICA-129Val* alleles have an increased risk to experience chronic GvHD (127). Furthermore, homozygotes *MICA-129Met* alleles carriers are at higher risk to experience relapse due to the exhaustion effect (121, 127). Despite the higher risk of relapse and aGvHD, patients with *Met/Met* have better overall survival than *Val/Val* carriers (121, 128). Martin et al. in 2020 implicated that patients receiving *MICA-129Met* graft have decreased risk of non-relapse mortality after HSCT (129). Another study describes a higher risk of CMV infection or reactivation and a higher risk of non-relapse mortality in patients receiving a graft from a donor having *MICA-129Val/Val* (130). The role of an isoform of *MICA-129* may also affect the serum level of soluble MICA (sMICA) (131). In vitro experiments show that MICA-129Met clones release more soluble MICA and a higher proportion of the *MICA-129Met* variant is retained in intracellular compartments which could influence HSCT outcome, but it needs to be confirmed in experiments using patients’ samples (131). The effect of soluble MICA on chronic GvHD development was described by Boukouaci et al. (127). The patients with sMICA > 80 pg/mL after transplantation have a higher risk of developing cGvHD than patients with sMICA < 80 pg/mL. Soluble MICA seems to be an even more important parameter than allele isoform of *MICA-129* as patients with sMICA > 80 pg/mL have always higher risk of cGvHD development regardless of *MICA-129* variant than patients with sMICA < 80 pg/mL (127).

### MICB and HSCT Outcome

Besides *MICA*, also *MICB* were observed to play a role in HSCT, as their match or mismatch seems to be a very important player in HSCT complications (124). Carapito et al. described the impact of mismatch of amino acid – isoleucine (Ile) and methionine (Met) – at position 98 within *MICB* (*MICB-98*) between donor and patient on GvHD and on overall survival (OS) affected by CMV (132). Although this mismatch occurs in approximately 6% of transplantations, such transplantation of hematopoietic stem cells from *MICB-98* mismatched but otherwise fully HLA and MICA matched donor increases risk of both acute and chronic GvHD development. By monitoring the effect of match/mismatch in *MICB-98* on overall survival affected by CMV, they found that *MICB-98* match significantly reduced the effect of CMV status on overall survival. Patients with matched graft had similar OS, regardless of CMV status. But when the patient received a mismatched graft and was CMV positive, there was fundamentally worse OS than for CMV negative patient with a mismatched graft (132). Match in *MICB-98* between patient and donor is then another parameter which would be good to monitor and which could help to select the optimal donor.

### ULBP and HSCT Outcome

Among ULBPs investigated, only the role of *ULBP6* in allogeneic stem cell transplantation has been described. Antoun et al. defined 2 common *ULBP6* alleles, ULBP6*001 or ULBP6*002. Patients with allele ULBP6*002 had better 8-year relapse-free survival (44% vs. 25%, p < 0.001) and better 8-year OS (55% vs. 39%, p = 0.003) than patients lacking this allele (102). Surprisingly, the protein of allele ULBP6*002 triggers lower cytotoxicity of NK cells. This seemingly controversial result can be explained by soluble ULBP6*002 which is attached to NKG2D with high affinity, disabling other NKG2D ligands to activate NK cells and limiting repeated triggering of NKG2D receptor (133, 134). Zuo et al. explain reduced survival after HSCT of carriers of ULBP6*001 variant by reduction of tumor antigen availability or elimination of antigen-presenting cells or T cells, which suppresses the subsequent development of alloreactive T cell immunity (133). These results need to be confirmed in further studies.

## Conclusions

As described, AML cells can deploy multiple protective mechanisms to survive and to spread. Part of these mechanisms involve NK cells and their recognition abilities. But these general mechanisms are not affecting all NK cells in the same way. Because NK activity is based on the balance of inhibitory and activating signals mediated by an interaction between ligands and receptors, AML cells downregulate some ligands for one of the most critical activating receptor NKG2D.

In various diseases, it has been described that polymorphisms of NKG2D ligands are positively associated with their development. For example, an allelic variant of MICA-129 was described to be associated with systemic lupus erythematosus (135), MICA*002 allele can have an effect in reducing the risk of primary sclerosing cholangitis (136), MICB*004 allele is associated with rheumatoid arthritis (137) and expression of *ULBP3* is upregulated in patients with alopecia areata (138). With all above associations and with first findings of the relationship between the different allelic distribution of selected NKG2D ligands in AML patients compared to healthy donors, we believe that individual polymorphisms could have a broader impact on AML development and progression. Still, more comprehensive studies should be done to draw relevant conclusions.

The importance of NKG2D’ and NKG2D ligands’ polymorphisms is also evident in studies that showed their correlation to patients’ outcome after HSCT. Additional markers may help to estimate the probability of post-transplant complications. Current data show that a match between patient and donor in MICA and in MICB can also be beneficial, but we still know very little about ULBP and about all polymorphisms playing a role in HSCT outcome. It becomes paramount to understand of the pathophysiological role of NKG2D and NKG2D ligands, and their role on HSCT outcome. This can be fundamental for donor selection to improve overall survival with strong GVL affect and low GvHD. This should be now feasible, with NGS sequencing becoming easier, more sensitive, and affordable for larger cohorts.

## Author Contributions

AM: planning and organizing structure of the review; research and analysis of the papers; and wrote the review. PP: planning and organizing structure of the review and contributions to the sections writing/critical review of the manuscript. MC: preparation of figures. MC, MH, VC, LH, and PJ: critical review of the manuscript. All authors contributed to the article and approved the submitted version.

## Funding

The work was funded by the Ministry of Health of the Czech Republic—Czech Health Research Council (project no. NV18-03-00277).

## Conflict of Interest

The authors declare that the research was conducted in the absence of any commercial or financial relationships that could be construed as a potential conflict of interest.
